# Cefepime–taniborbactam and ceftibuten–ledaborbactam maintain activity against KPC variants that lead to ceftazidime–avibactam resistance

**DOI:** 10.1128/aac.01511-24

**Published:** 2025-02-10

**Authors:** Cullen L. Myers, Annie Stevenson, Brittany Miller, Denis M. Daigle, Tsuyoshi Uehara, Daniel C. Pevear

**Affiliations:** 1 Department of Molecular and Cellular Biology, University of Guelph 317113 , Guelph, Ontario, Canada; 2 Venatorx Pharmaceuticals Inc. 540451 , Malvern, Pennsylvania, USA; University of Pittsburgh School of Medicine, Pittsburgh, Pennsylvania, USA

**Keywords:** taniborbactam, ledaborbactam, KPC, β-lactamase inhibitor, ceftazidime–avibactam, antibiotic resistance

## Abstract

*Klebsiella pneumoniae* carbapenemases (KPCs) are widespread β-lactamases that are a major cause of clinical non-susceptibility of Gram-negative bacteria to carbapenems and other β-lactam antibiotics. Ceftazidime combined with the β-lactamase inhibitor avibactam (CAZ–AVI) has been effective in treating infections by KPC-producing bacteria, but emerging KPC variants confer resistance to the combination. Taniborbactam and ledaborbactam are bicyclic boronate β-lactamase inhibitors currently under development with cefepime and ceftibuten, respectively, to treat carbapenem-resistant bacterial infections. Here, we assessed the effects of clinically important KPC-2 and KPC-3 variants (V240G, D179Y, and D179Y/T243M) on the antibacterial activity of cefepime–taniborbactam (FEP–TAN) and ceftibuten–ledaborbactam (CTB–LED) and examined catalytic activity and inhibition of these variants. Unlike CAZ–AVI, FEP–TAN and CTB–LED were highly active against *Escherichia coli* strains expressing these KPC variants. Experiments with purified enzymes showed that FEP and CTB were poorly hydrolyzed by the KPC variants and had weak affinity for variants containing D179Y. In addition, the D179Y substitution in KPC-2 reduced inhibition by TAN and LED, but inactivation efficiencies (*k*_2_/*K*) for these inhibitors were significantly higher than those for AVI. *K*_2_/*K* was less affected for D179Y-containing KPC-3 variants, and robust inhibition was observed by TAN, LED, and AVI. Together, the findings illustrate a biochemical basis for FEP–TAN and CTB–LED efficacy in KPC variant-mediated CAZ–AVI resistance backgrounds, whereby the boronate inhibitors have sufficient inhibitory activity, while FEP and CTB are poor substrates and bind to the variant enzymes with reduced affinity.

## INTRODUCTION

β-Lactam (BL) antibiotics continue to be the first-line therapy for the treatment of Gram-negative bacterial infections due to their proven track record of clinical safety and efficacy (1). Among the various mechanisms of bacterial resistance to these drugs, the production of β-lactamase enzymes that hydrolyze the BL ring is foremost (1). β-Lactamases are grouped into four classes—serine β-lactamases (classes A, C, and D) that use a nucleophilic serine to catalyze BL hydrolysis and class B metallo β-lactamases that rely on active site zinc ions (2). Of particular concern are the globally disseminating *Klebsiella pneumoniae* carbapenemases (KPCs). These enzymes inactivate virtually all β-lactam antibiotics, which limits therapeutic options for infections by KPC-producing bacteria, making such infections a serious global health threat (3, 4).

To address β-lactamase-mediated antibiotic resistance, β-lactamase inhibitors (BLIs) have been deployed to the clinic, with demonstrable success at rescuing BL efficacy in the treatment of infections by β-lactamase-producing bacteria (5). KPCs, however, are refractory to early-generation BLIs that possess the BL moiety, e.g., clavulanic acid and tazobactam (6, 7). This spurred the development of BLIs derived from non-BL scaffolds that achieve clinically relevant inhibition of KPCs (8, 9). The combination of ceftazidime with the diazabicyclooctane BLI avibactam (CAZ–AVI) received FDA approval in 2015 (10, 11), and in 2017, the first (mono)cyclic boronate BLI, vaborbactam (VAB), was approved for clinical use in combination with meropenem (MEM) (12).

CAZ–AVI was a welcome introduction to the clinic, but concurrent with and following its approval for clinical use, the combination was shown to select for CAZ–AVI resistance linked to *bla*_KPC_ mutations *in vitro* (13, 14). The Ω-loop region of the protein, which spans residues 162–179 in KPC, has proved to be a hotspot for amino acid changes resulting from such mutations. A prime example is substitution at D179 (13, 15, 16), but mutations causing amino acid substitutions outside the Ω-loop, e.g., V240G, have also been associated with reduced CAZ–AVI efficacy (17). Worryingly, KPC variants linked to CAZ–AVI resistance have since emerged during the course of CAZ–AVI therapy (18, 19). Moreover, variants with substitutions at D179 poorly hydrolyze carbapenems, which complicates treatment since infections initially present as MEM-resistant, but revert to MEM-susceptible while becoming CAZ–AVI-resistant following treatment with the combination (19–21). Substitutions at D179 also reportedly directly affect the inhibitory activity of AVI and VAB, the latter being less affected (20–22).

Taniborbactam (TAN) is a bicyclic boronate BLI being developed with cefepime (FEP) for the treatment of complicated urinary tract infections, as well as hospital- and ventilator-associated bacterial pneumonia infections caused by β-lactamase-producing Enterobacterales and *Pseudomonas aeruginosa* (23–25). Ledaborbactam (LED) is the active form of the orally bioavailable bicyclic boronate prodrug ledaborbactam-etzadroxil that is in development with ceftibuten (CTB) to address multidrug-resistant β-lactamase-expressing Enterobacterales (26, 27). TAN and LED both provide excellent coverage of serine β-lactamases (including KPCs), and the inhibition spectrum for TAN encompasses clinically relevant variants of the metallo β-lactamases NDM and VIM (24). In this study, we compared the antibacterial activity of FEP–TAN, CTB–LED, and CAZ–AVI in isogenic *E. coli* strains producing KPC-2 and KPC-3 variants implicated in CAZ–AVI resistance and examined the catalytic properties and inhibition of these variants.

## RESULTS

### Effect of KPC variant expression on antibacterial activity

Table 1 shows the minimum inhibitory concentrations (MICs) for BLs and BL–BLI combinations against isogenic *E. coli* strains expressing CAZ–AVI-resistant KPC-2 or KPC-3 variants (V240G: KPC-6, KPC-8; D179Y: KPC-31, KPC-32; and D179Y/T243M: KPC-33). KPC-2^V240G^ increased the MICs for BLs relative to the control strain lacking KPC expression, and while TAN, AVI, or LED fully restored FEP or CTB MICs, CAZ–AVI and CAZ-TAN MICs remained elevated at 4–8 µg/mL. Owing to previously described collateral sensitivity towards carbapenems (14, 19), MICs for MEM were modestly increased (eightfold) by the expression of KPC-2^D179Y^ or KPC-2^D179Y/T243M^ relative to the control strain compared with increases of >512-fold for CAZ, 64-fold for FEP, and 8–16-fold for CTB. MICs for CAZ–AVI remained highly elevated (>256 µg/mL), but FEP–TAN and CTB–LED were active at ≤0.5 µg/mL. FEP–AVI was active against these strains at MICs of 2 and 0.5 µg/mL, respectively, possibly reflecting greater FEP stability to the KPC variants compared with CAZ. Indeed, FEP alone was active at 16 µg/mL compared with >256 µg/mL for CAZ, and TAN rescued CAZ MICs to just 16 µg/mL.

**TABLE 1:** Antibacterial activity (MIC, μg/mL) of BLs and BL/ BLIi^*a*^ combinations against engineered *E. coli* strains expressing KPC-2^*b*^ and KPC-3*^b^* variants relative to a control strain lacking a β-lactamase

KPC variant expressed	MEM	CAZ	CAZ + AVI	CAZ + TAN	FEP	FEP + TAN	FEP + AVI	CTB	CTB + LED
None	0.03	0.5	0.5	0.5	0.12	0.12	0.12	1	0.25
KPC-2									
wt	32	64	1	0.5	64	0.12	0.25	16	0.25
V240G	8	>256	8	4	128	0.25	0.25	64	0.5
D179Y	0.25	>256	64	16	16	0.5	2	16	0.5
D179Y/T243M	0.25	>256	64	16	16	0.25	0.5	16	0.25
KPC-3									
wt	16	256	4	2	128	0.25	0.5	32	0.5
V240G	16	>256	128	16	256	0.5	1	32	0.5
D179Y	0.12	>256	256	32	32	1	4	32	1
D179Y/T243M	0.12	>256	256	32	16	0.5	2	16	0.5

^*a*^ BLs were tested with BLIs at a fixed concentration of 4 μg/mL.

^*b*^ KPC-2 and KPC-3 differ by a single amino acid: H274 in KPC-2, Y274 in KPC-3. Abbreviations: MEM, meropenem; CAZ, ceftazidime; AVI, avibactam; TAN, taniborbactam; FEP, cefepime; CTB, ceftibuten; LED, ledaborbactam.

The MIC for CAZ–AVI was elevated 256-fold against the KPC-3^V240G^-expressing strain relative to the control, whereas TAN and LED fully restored FEP or CTB activity. Against this strain, TAN only partially rescued CAZ (MIC of 16 µg/mL), while FEP activity was rescued to an MIC of 1 µg/mL by AVI. Strains expressing D179Y-containing KPC-3 variants had highly elevated CAZ–AVI MICs (256 µg/mL), but FEP–TAN and CTB–LED MICs were 0.5–1 µg/mL. Although AVI achieved appreciable rescue of FEP against these strains with MICs of 0.5–4 µg/mL, CAZ–TAN activity was markedly weaker at 16 µg/mL. Again, CAZ MICs in the absence of a BLI were significantly higher than FEP or CTB for these strains.

Overall, these results confirm our previous reporting of potent antibacterial activity for FEP–TAN or CTB–LED against CAZ–AVI-resistant KPC variant-producing strains (24, 27) and allude to different effects of the substitutions on the hydrolytic activity and inhibition of KPC-2 versus KPC-3.

### Catalytic activity of KPC variants

We next determined kinetic parameters for BL substrate hydrolysis using purified KPC enzymes, shown in Table 2. Kinetic parameters for all substrates tested with KPC-2^V240G^ were comparable to KPC-2^wt^. In contrast to linear kinetics for CAZ hydrolysis by KPC-2^wt^ and KPC-2^V240G^, D179Y-containing KPC-2 variants displayed saturation kinetics for CAZ hydrolysis, with reduced *K*_M_ and increased *k*_cat_/ *K*_M_ relative to KPC-2^wt^, consistent with previous findings (20, 22). *K*_M_ for FEP hydrolysis was also reduced with these variants, but unlike CAZ, catalytic efficiency (*k*_cat_/*K*_M_) decreased relative to wild-type KPC-2, and both variants poorly hydrolyzed CTB. Interestingly, KPC-2^D179Y^ or KPC-2^D179Y/T243M^ hydrolyzed FEP with a higher *k*_cat_ /*K*_M_ than CAZ, yet FEP had lower MICs against strains expressing these variants (Table 1). Still, the findings are in agreement with reports of elevated CAZ MICs against strains expressing KPC variants with D179 substitutions that appeared to have reduced hydrolytic activity against this substrate (20–22).

**TABLE 2:** Kinetic parameters for β-lactam hydrolysis by KPC variants^*a*^

Variant and parameter	WT	V240G	D179Y	D179Y/T243M
KPC-2				
CENTA (28)				
*K*_M_ (µM)	19.7 ± 1.3	63.3 ± 2.3	1.9 ± 0.2	2.1 ± 0.3
*k*_cat_ (s^−1^)	243 ± 11	662 ± 9.6	(1.2 ± 0.08) × 10^−2^	(0.9 ± 0.04) × 10^−2^
*k*_cat_/*K*_M_ (s^−1^⋅M^−1^)	(1.5 ± 0.07) × 10^7^	(4.1 ± 0.06) × 10^7^	(7.4 ± 0.5) × 10^2^	(5.5 ± 0.3) × 10^5^
Ceftazidime				
*K*_M_ (µM)	>500	>500	0.6 ± 0.09	1.0 ± 0.2
*k*_cat_ (s^−1^)	ND	ND	(1.2 ± 0.04) × 10^−3^	(3.7 ± 0.13) × 10^−3^
*k*_cat_/*K*_M_ (s^−1^⋅M^−1^)	(7.3 ± 1.3) × 10^2^	(8.0 ± 0.33) × 10^2^	2.0 × 10^3^	3.7 × 10^3^
Cefepime				
*K*_M_ (µM)	>500	>500	3.7 ± 0.7	3.0 ± 0.6
*k*_cat_ (s^−1^)	ND	ND	(1.5 ± 0.07) × 10^−2^	(1.7 ± 0.06) × 10^−2^
*k*_cat_/*K*_M_ (s^−1^⋅M^−1^)	(1.3 ± 0.04) × 10^5^	(1.9 ± 0.3) × 10^5^	4.0 × 10^3^	5.7 × 10^3^
Ceftibuten				
*K*_M_ (µM)	>500	>500	ND	ND
*k*_cat_ (s^−1^)	ND	ND	ND	ND
*k*_cat_/*K*_M_ (s^−1^⋅M^−1^)	(2.2 ± 0.24) × 10^3^	(2.0 ± 0.25) × 10^3^	ND	ND
KPC-3				
CENTA				
*K*_M_ (µM)	105 ± 24	54.8 ± 8.3	124 ± 17	153 ± 25
*k*_cat_ (s^−1^)	38 ± 3.4	12.7 ± 1.1	0.24 ± 0.02	0.18 ± 0.02
*k*_cat_/*K*_M_ (s^−1^⋅M^−1^)	(3.7 ± 0.5) × 10^5^	(2.3 ± 0.2) × 10^5^	(1.2 ± 0.1) × 10^3^	(1.2 ± 0.07) × 10^3^
Ceftazidime				
*K*_M_ (µM)	>500	>500	15.5 ± 4.7	14.6 ± 4.1
*k*_cat_ (s^−1^)	ND	ND	1.2 ± 0.1	1.6 ± 0.2
*k*_cat_/*K*_M_ (s^−1^⋅M^−1^)	(2.3 ± 0.19) × 10^3^	(3.8 ± 0.05) × 10^3^	(7.9 ± 1.7) × 10^4^	(1.1 ± 0.2) × 10^5^
Cefepime				
*K*_M_ (µM)	>500	>500	90 ± 31	42.5 ± 12.5
*k*_cat_ (s^−1^)	ND	ND	38 ± 7	18.7 ± 2.3
*k*_cat_/*K*_M_ (s^−1^⋅M^−1^)	(4.2 ± 0.04) × 10^4^	(4.2 ± 0.13) × 10^4^	(4.2 ± 0.7) × 10^5^	(4.4 ± 0.8) × 10^5^
Ceftibuten				
*K*_M_ (µM)	>500	>500	ND	ND
*k*_cat_ (s^−1^)	ND	ND	ND	ND
*k*_cat_/*K*_M_ (s^−1^⋅M^−1^)	(0.95 ± 0.09) × 10^3^	(1.3 ± 0.13) × 10^3^	ND	ND

^*a*^ Data are reported as the mean ± standard deviation (*n* = 3). *k*_cat_/ *K*_M_ was determined from plots of velocity versus substrate concentration, where [*S*]<< *K*_M_ (29). ND, not determined.

Like KPC-2, kinetic parameters for substrate hydrolysis by KPC-3^V240^ were similar to KPC-3^wt^ (Table 2). *K*_M_s for CENTA hydrolysis by KPC-3^D179Y^ and KPC-3^D179Y/T243M^ were comparable to KPC-3^wt^, but *k*_cat_ was significantly reduced. Additionally, D179Y-containing KPC-3 variants displayed saturation kinetics for CAZ and FEP hydrolysis with reduced *K*_M_s but increased *k*_cat_/ *K*_M_ relative to KPC-3^wt^. Indeed, these variants hydrolyzed CAZ with markedly lower *K*_M_ and *k*_cat_ than FEP.

**Fig 1:**
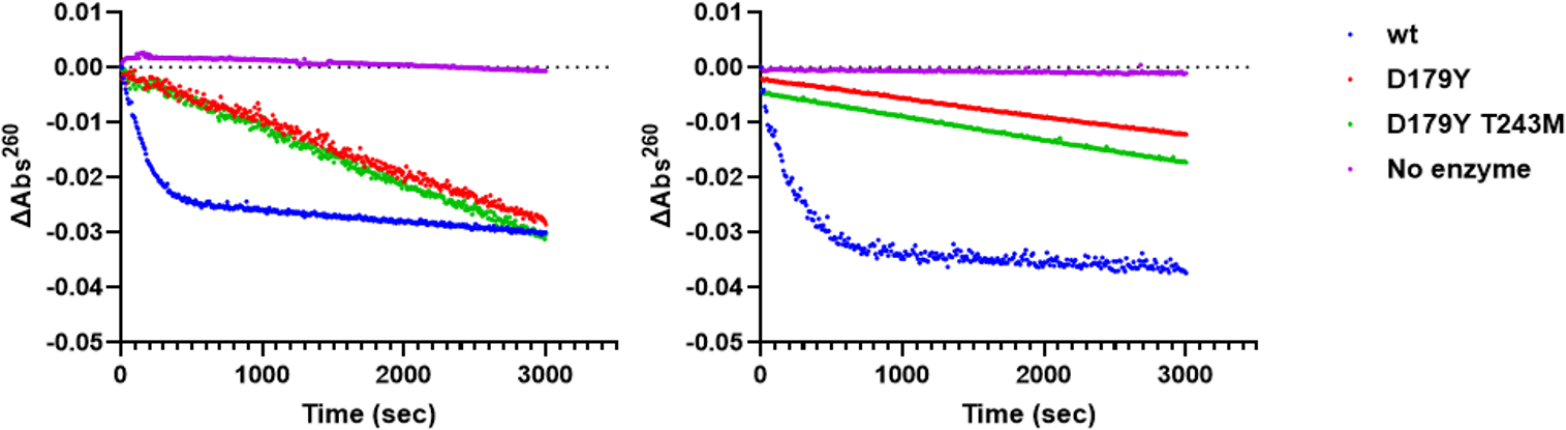
Hydrolysis of CTB by KPC variants. Progress curves are shown for reactions that contained 20 µM CTB and 1 µM of KPC-2 (left) or KPC-3 (right) variants.

Even though CTB MICs against strains expressing D179Y-containing KPC variants were elevated (Table 1), kinetic parameters for CTB hydrolysis could not be determined under the assay conditions. Progress curves confirmed these variants hydrolyzed CTB, albeit with significantly slower turnover than the wild-type enzymes (Fig. 1) – CTB hydrolysis neared completion within five minutes for wild-type enzymes, compared to ~50 minutes for KPC-2 D179Y-containing variants and 30 – 40% CTB conversion by D179Y-containing KPC-3 variants within the same timeframe (Fig. 1).

A reduction in substrate turnover coupled with an apparent increase in affinity (i.e. reduced *K*_M_), may indicate slow product release relative to the formation of enzyme-substrate complexes. Hence, the affinities of CAZ, FEP and CTB for D179Y-containing KPC variants were assessed by determining their apparent *K*_i_ for inhibition of CENTA hydrolysis (Table 3). CAZ and FEP showed significantly higher affinity for D179Y-containing KPC-2 variants relative to the wild-type enzyme, but FEP exhibited ~10 fold weaker affinity for the variants than CAZ. The apparent affinity of FEP for KPC-3^wt^ was comparable to that of CAZ, but FEP demonstrated weaker affinity for the D179Y-containing KPC-3 variants relative to KPC-3^wt^. Notably, CTB demonstrated weak affinity for wild-type KPC enzymes and lacked measurable inhibition of CENTA hydrolysis by KPC-2/KPC-3 D179Y-containing variants.

**TABLE 3:** Apparent affinities (*K*_i_^app^, µM) of CAZ, FEP and CTB for KPC variants^*a*^

KPC variant	CAZ	FEP	CTB
KPC-2			
wt	19.8 ± 3.0	32.9 ± 5.6	159 ± 10
D179Y	0.55 ± 0.12	4.6 ± 1.2	ND
D179Y/ T243M	0.60 ± 0.19	3.2 ± 0.6	ND
KPC-3			
wt	437 ± 184	276 ± 47	368 ± 26
D179Y	90 ± 3.1	1041 ± 72	ND
D179Y/T243M	177 ± 27	728 ± 58	ND

^*a*^ Data are reported as the mean ± standard deviation (*n* = 3). ND, no significant inhibition of CENTA hydrolysis was detected at the highest concentration tested.

### Inhibition of KPC variants

The data presented in Tables 2 and 3 suggest that the microbiological activities observed against KPC variant-producing strains result from differences in the biochemical interactions between the variants and the BL substrates. However, the microbiological data could be interpreted as indicating differing abilities of the BLIs to inhibit the KPC variants. For instance, TAN and LED rescued partner cephalosporins within 1–2 dilutions of the MICs for control strains, whereas MICs in the presence of AVI were invariably higher (see Table 1). Therefore, to delineate contributions from KPC variant inhibition, kinetic constants for inhibition of the variants were determined.

AVI inactivated KPC V240G variants with reduced efficiencies (*k*_2_/*K*) compared to the wild-type enzyme (Table 4). Dissociation rates were not impacted, and thus AVI *K*_d_ was increased for these variants. Similar observations were noted for TAN inhibition of KPC-2^V240G^. The *K*_d_ of TAN was also higher for KPC-3^V240G^ relative to the wild-type enzyme, which was in this case due to a faster dissociation rate since *k*_2_/*K* was not impacted. These findings support the MIC results herein, along with previous results that showed TAN and LED restored FEP or CTB antibacterial activity against *E. coli* expressing KPC-3^V240G 24,27^.

**TABLE 4:** Kinetic parameters for inhibition of KPC variants^*a*^

Variant and BLI	Parameter	Wt	V240G	D179Y	D179Y/T243M
KPC-2					
AVI	*k*_2_/*K* (M^−1^⋅s^−1^)	(3.6 ± 0.2) × 10^4^	(1.2 ± 0.1) × 10^4^	2.0 ± 0.06	4.0 ± 0.1
	*k*_off_ (s^−1^)	(5.3 ± 0.03) × 10^−4^	(5.4 ± 0.04) × 10^−4^	(5.7 ± 0.6) × 10^−5^	(5.8 ± 0.2) × 10^−5^
	*t*_1/2_ (min)	21.4 ± 0.07	21.5 ± 0.06	205 ± 10	198 ± 8
	*K*_d_ (µM)	0.015	0.045	28.3	14.6
TAN	*k*_2_/*K* (M^−1^⋅s^−1^)	(0.9 ± 0.02) × 10^4^	(0.2 ± 0.02) × 10^4^	17 ± 0.7	15.2 ± 0.1
	*k*_off_ (s^−1^)	(1.0 ± 0.04) × 10^−4^	(1.1 ± 0.03) × 10^−4^	(5.0 ± 0.4) × 10^−5^	(5.2 ± 0.3) × 10^−5^
	*t*_1/2_ (min)	104 ± 2.3	106 ± 2.3	231 ± 19.4	222.3 ± 13.7
	*K*_d_ (µM)	0.011	0.055	2.9	3.4
LED^*b*^	*k*_2_/*K* (M^−1^⋅s^−1^)	(2.9 ± 0.07) × 10^4^	(3.1 ± 0.8) × 10^4^	(5.2 ± 0.6) × 10^2^	(4.3 ± 0.3) × 10^2^
	*k*_off_ (s^−1^)	(2.5 ± 0.1) × 10^−4^	(3.7 ± 0.1) × 10^−4^	(6.9 ± 0.2) × 10^−4^	(6.3 ± 1.0) × 10^−4^
	*t*_1/2_ (min)	46 ±2	32 ± 1	16.7 ± 0.5	18.6 ± 2.7
	*K*_d_ (µM)	0.009	0.012	1.3	1.5
KPC-3					
AVI	*k*_2_/*K* (M^−1^⋅s^−1^)	(4.0 ± 0.3) × 10^4^	(2.4 ± 0.4) × 10^4^	(7.0 ± 0.5) × 10^3^	(4.6 ± 0.8) × 10^3^
	*k*_off_ (s^−1^)	(1.1 ± 0.009) × 10^−3^	(1.1 ± 0.009) × 10^−3^	(9.0 ± 0.7) × 10^−4^	(5.2 ± 0.7) × 10^−4^
	*t*_1/2_ (min)	10.1 ± 0.1	10.7 ± 0.1	12.8 ± 0.9	22.5 ± 3.5
	*K*_d_ (µM)	0.028	0.046	0.129	0.113
TAN	*k*_2_/*K* (M^−1^⋅s^−1^)	(3.9 ± 0.4) × 10^4^	(4.4 ± 0.9) × 10^4^	(2.8 ± 0.2) × 10^3^	(4.4 ± 0.7) × 10^3^
	*k*_off_ (s^−1^)	(3.5 ± 0.4) × 10^−4^	(8.0 ± 0.4) × 10^−4^	(8.1 ± 0.7) × 10^−4^	(8.5 ± 2.5) × 10^−4^
	*t*_1/2_ (min)	34 ± 4	14.4 ± 0.7	14.3 ± 1.2	14.6 ± 5
	*K*_d_ (µM)	0.009	0.018	0.29	0.193
LED	*k*_2_/*K* (M^−1^⋅s^−1^)	(2.7 ± 0.05) × 10^3^	(1.3 ± 0.4) × 10^4^	(5.4 ± 2.4) × 10^2^	(1.0 ± 0.3) × 10^3^
	*k*_off_ (s^−1^)	(5.9 ± 0.06) × 10^−4^	(6.0 ± 0.1) × 10^−4^	(6.7 ± 0.1) × 10^−4^	(6.5 ± 0.8) × 10^−4^
	*t*_1/2_ (min)	19.6 ± 0.2	19.3 ± 0.4	17.2 ± 0.3	18.1 ± 2.2
	*K*_d_ (µM)	0.22	0.046	1.25	0.62

^*a*^ Data are reported as the mean ± standard deviation (*n* = 3).

^*b*^ Kinetic data for ledaborbactam with KPC-2^wt^ wre previously reported (27).

In agreement with Compain and Arthur (20), *k*_2_/*K* for AVI was reduced 18,000-fold with KPC-2^D179Y^ and 9,000-fold with KPC-2^D179Y/T243M^ (Table 4). Inactivation efficiencies of KPC-2^D179Y^ and KPC-2^D179Y/T243M^ by TAN were also reduced, but to a much lesser degree – roughly 500- and 600-fold, respectively. Estimations of *K*_d_ indicated TAN had higher affinity than AVI for these variants. Inhibition of KPC-2^D179Y^ and KPC-2^D179Y/T243M^ by LED was even less impacted than TAN, with ~60 fold and ~70 fold reductions in *k_2_/K* for KPC-2^D179Y^ and KPC-2^D179Y/T243M^, respectively, and improved *K*_d_s compared to AVI or TAN. These findings confirm that TAN and LED inhibitory activities are less affected by the D179Y substitution in KPC-2, which contributes to greater rescue of the antimicrobial activity by the partner BL compared to AVI (e.g. rescue of CAZ activity by TAN, Table 1).

TAN, LED and AVI inactivated KPC-3^D179Y^ and KPC-3^D179Y/T243M^ with modestly reduced (~2–14 fold) efficiencies relative to KPC-3^wt^ (Table 4). Similar findings for AVI have been reported by Shapiro and colleagues (30). Dissociation kinetics for TAN, AVI and LED were faster with D179Y-containing KPC-3 variants than corresponding KPC-2 variants, and while AVI and TAN underwent slower dissociation from KPC-2^D179Y^ variants compared to KPC-2^wt^, off-rates for KPC-3 D179Y variants were similar to KPC-3^wt^. Therefore, the D179Y substitution produced different impacts on the inhibition of KPC-3 versus KPC-2.

### Molecular basis for the impact of the D179Y substitution on inhibition of KPC

The finding that the D179Y mutation caused different effects on the inhibitory properties of TAN, LED and AVI underscored distinct modes of inhibitor binding to these variants. X-ray crystal structures have been solved for complexes of AVI (31) and TAN (32) with KPC-2. Both inhibitors engage active site residues S130, N132, T235 and T237. Additionally, W105, thought to be important for β-lactam substrate recognition (33), makes modest van der Waals interactions with the 6-membered piperidine ring in AVI and participates in hydrophobic interactions with bicyclic boronate core in TAN (31, 32).

**Fig 2:**
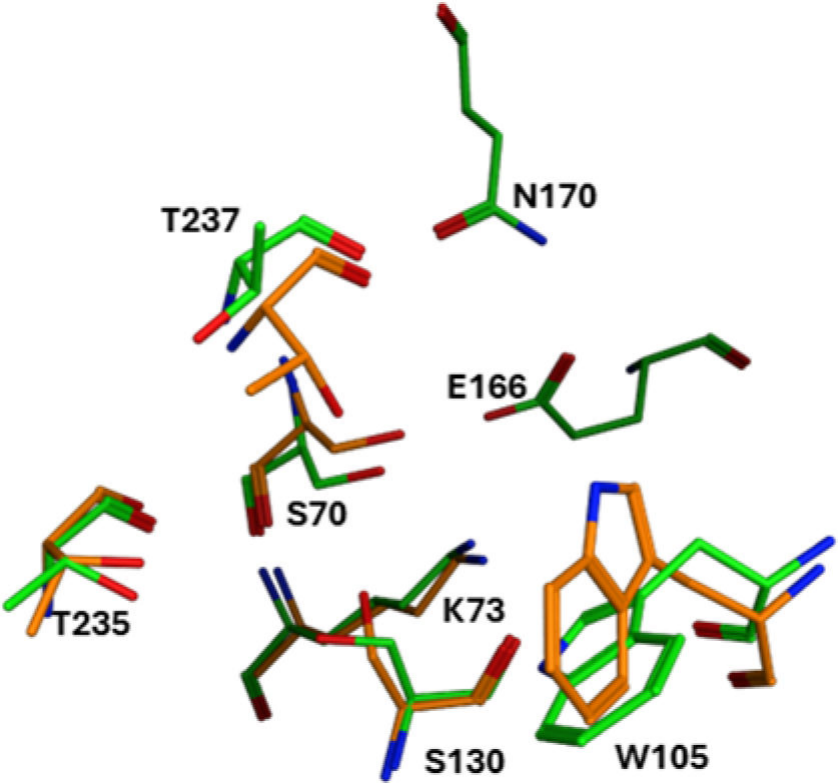
Comparison of active site residues for KPC-2^wt^ (green) and KPC-2^D179Y^ (orange).

The recently determined crystal structure for KPC-2^D179Y^ showed little deviation in the orientation of key active site residues, except for W105 which was flipped perpendicular to its orientation in KPC-2^wt^ (17) (Fig. 2). Also, the absence of electron density for Ω-loop residues in the KPC-2^D179Y^ structure was consistent with a disordered Ω-loop, which has been further supported by NMR spectroscopy findings (34). Accordingly, the loss of inhibitor interactions dependent on the loop would likely account for the diminished inhibition of this variant.

**Fig 3:**
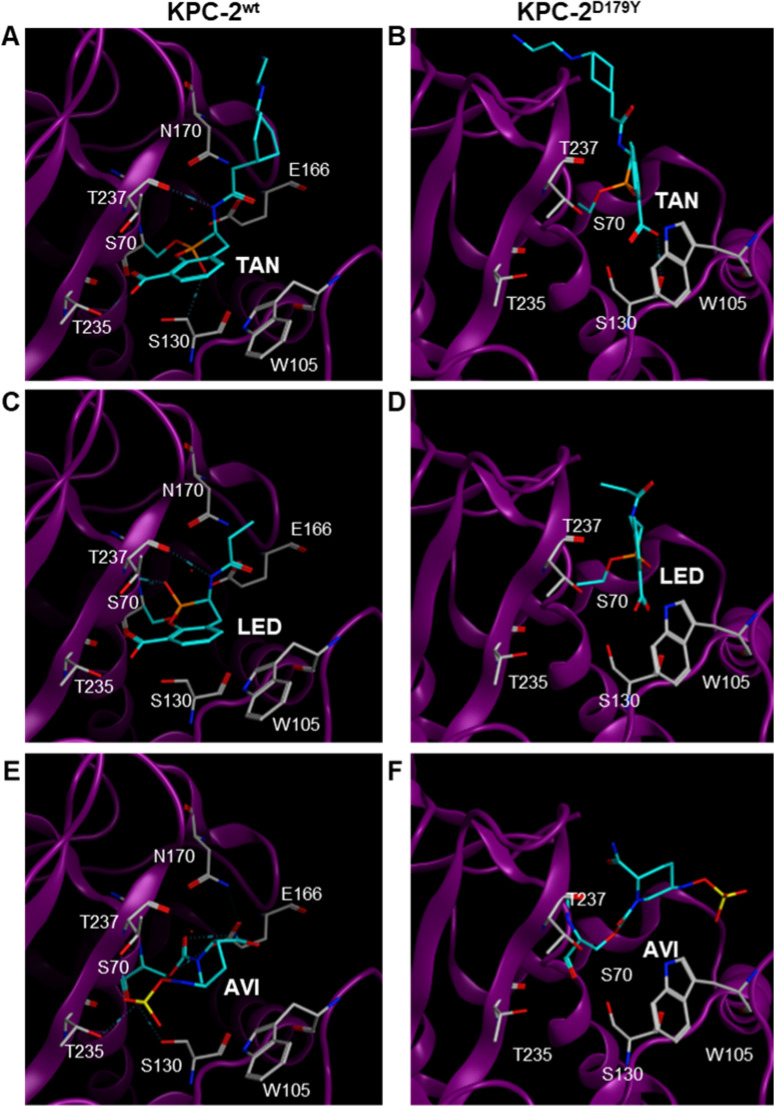
Molecular docking of TAN, LED and AVI with KPC-2^wt^ (A, C and E) and KPC-2^D179Y^ (B, D and F).

Covalent docking of TAN to KPC-2^D179Y^ implied that critical interactions with T235 and T237 could be compromised (Fig. 3A and B). It has been suggested that the disorder of the Ω-omega loop in this variant would be transmitted to the region containing W105 (33), thus perturbing inhibitor interactions with this residue. Accordingly, the bicyclic core of TAN appeared displaced from its interaction with W105 (Fig. 3B). For AVI, interactions with W105 in KPC-2^D179Y^ are completely lost, along with the interaction with S130 (Fig. 3E and F ). Modelling of LED in the KPC-2^wt^ active site showed its bicyclic core engaged in interactions similar to those observed with TAN, and interactions with T235, T237 and especially W105 in KPC-2^D179Y^ were disrupted in an analogous manner (Fig. 3C and D ).

## DISCUSSION

Prior to the introduction of CAZ–AVI, treatment options for infections by carbapenemase-expressing Gram-negative bacteria were limited to less desirable agents with unfavourable toxicity or efficacy profiles. Chief among such carbapenemases are KPCs, but KPC variant-mediated CAZ–AVI resistance has quickly evolved in the clinic. *Bla*_KPC_ mutations causing amino acid changes in the Ω-loop, especially at D179, were among the first and most commonly identified, and additional variants are being identified with more frequency (35). In this study, we showed that bicyclic boronate BLIs TAN and LED restored cephalosporin activity against engineered CAZ–AVI-resistant *E. coli* strains expressing KPC-variants by circumventing distinct effects on the hydrolytic activity and inhibition of KPC-2 versus KPC-3.

A key finding from this work was that the hydrolysis and interactions of CAZ were more profoundly affected than FEP or CTB. Of the three variants, KPC-2/KPC-3 possessing the V240G substitution caused the least resistance to CAZ–AVI. V240 is in a region adjacent to the Ω-loop and changes at this site have been proposed to affect Ω-loop dynamics (17). However, it was apparent that the highest concentrations of CAZ, FEP or CTB used in MICs assays (256 µg/mL) would result in intra-cellular concentrations well below the respective *K*_M_s for V240G-containing variants, or corresponding wild-type enzymes (see Table 2). Hence, these enzymes would be operating below maximal efficiency, which would enable BLI rescue in cells. Even so, it was notable that FEP or CTB activity was more effectively rescued than CAZ in a V240G variant expression background, likely due to a higher *k*_cat_ for CAZ by these variants.

Effects from substitutions at D179 on CAZ hydrolysis by KPC, and the antibacterial activity of CAZ or CAZ–AVI, have been studied previously (17, 21, 22, 30). This residue is engaged in a salt bridge with R164 that stabilizes the Ω-loop, and loop disorder resulting from substitutions at D179 is thought to allow for better accommodation of CAZ in the active site, enhancing its interaction with the enzyme (21). This aligns with the reductions in *K*_M_ (and presumably *k*_cat_) observed here for the D179Y-containing variants with CAZ, as well as with FEP, and is consistent with the prolonged existence of the acyl-enzyme complex (21, 36). Our observations therefore affirm the proposed role of increased substrate affinity in CAZ–AVI resistance mediated by KPC^D179Y^ or KPC^D179Y/T243M^. Importantly, FEP bound D179Y-containing variants with significantly weaker affinity than CAZ, while CTB lacked measurable affinity by the approaches used here. This emphasizes the influence of BL molecular structure (particularly the presence and nature of the R2 group) on binding to KPC variants.

Molecular docking experiments suggested that the loss of key hydrophobic interactions with W105 could possibly explain reduced inhibition of KPC-2^D179Y^, and the relative potency of the BLIs. Like TAN, LED took longer to form a covalent adduct with KPC-2^D179Y^ than with the wild-type enzyme, but KPC-2^D179Y^ was more efficiently inactivated by LED than by TAN. A possible explanation could be that the propionamide moiety in LED experiences less steric hindrance from Y179 compared to the cyclohexyl diamine in TAN (or the piperidine ring in AVI). Additionally, the inherent flexibility of the cyclohexyl diamine in TAN may hinder adduct formation to KPC-2^D179Y^. This could be exacerbated by the disordered Ω-loop in KPC^D179Y^ that may encourage unfavourable steric clashes with the cyclohexyl diamine in TAN (or the piperidine ring in AVI).

We have shown here that KPC-2 and KPC-3 variants exhibited different catalytic properties with respect to BL hydrolysis and inhibition, but it is not immediately obvious why this is the case. The two enzymes differ at a single position – residue 274 is a histidine in KPC-2 and a tyrosine in KPC-3. It has been suggested that Y274 in KPC-3 supplies additional interactions to CAZ (29), which can be envisioned to apply to inhibitor binding. Alternatively, this residue could modulate protein dynamics with implications for substate/ inhibitor binding. Additional biochemical, structural and biophysical studies are needed to elucidate the details underlying the different effects these resistance-conferring amino acid substitutions on the properties of KPC-2 versus KPC-3.

### Summary and conclusions

FEP–TAN and CTB–LED maintain activity against isogenic *E. coli* strains expressing KPC variants that confer resistance to CAZ–AVI. Biochemical studies revealed that sufficient inhibition by the boronate inhibitors along with weak hydrolysis and poorer affinity of the partner cephalosporins enable FEP–TAN and CTB–LED efficacy against strains producing KPC-2 variants. FEP–TAN and CTB–LED activity against KPC-3 variant-producing strains, however, is due primarily to the weaker affinity of these enzymes for FEP or CTB compared to CAZ, and poor hydrolysis of CTB. Overall, the findings show that strains expressing KPC variants of clinical importance are within the spectrum of antibacterial activity for the cephalosporin-bicyclic boronate BLI combinations and offer evidence supporting their use for treating infections caused by KPC-producing Enterobacterales.

## MATERIALS AND METHODS

### Bacterial strains and microbiological assays

Isogenic strains were engineered in *E. coli* DH5α. DNA sequences encoding the periplasmic domain of KPC variants, preceded by a signal sequence to achieve periplasmic localization, were placed under control of the *bla*_TEM-1_ promoter then cloned into plasmid pTwist Chlor High Copy in *E. coli* DH5α as previously described (24). MICs were determined by broth microdilution according to CLSI standard methods (37, 38).

### Protein expression and purification

DNA sequences encoding full-length KPC-2, and variants were cloned into pET9A plasmid and plasmid constructs were used to transform *E. coli* BL21 (DE3) cells. Cells were cultured in LB medium supplemented with 35 µg/mL kanamycin to an OD_600_ of 0.6–0.8, after which cultures were cooled, then protein expression induced with 1 mM isopropyl-β-D-1-thiogalactopyranoside (IPTG) for 16 hours at 18°C. Cells were harvested by centrifugation at 6,000 × g and the periplasmic contents, containing mature KPC-2 proteins, were extracted by cold osmotic shock (39) in the presence of 0.02 mg/mL lysozyme. KPC-2 proteins were purified using ion-exchange chromatography, followed by size-exclusion chromatography, as previously described (24).

The sequence encoding the mature, periplasmic domain of KPC-3 and variants (residues 27–293) were cloned into pET28a plasmid and the resulting constructs transformed into *E. coli* BL21 (DE3) cells. Cells were grown at 37°C in 1 L of LB medium supplemented with 35 µg/mL kanamycin to an OD_600_ of 0.6–0.8, after which cultures were cooled, then protein expression was induced with 1 mM IPTG for 16 hours at 18°C. Cells were harvested by centrifugation (6,000 × g), resuspended in buffer A (20 mM Tris [pH 7.5], 300 mM NaCl, 5% glycerol) supplemented with EDTA-free protease inhibitor, then lysed by sonication (Branson Sonifier 250). Lysates were clarified by centrifugation at 14,000 × g, and KPC-3 proteins purified using immobilized metal ion chromatography, by applying lysates to 5 mL of Ni-NTA resin (ThermoFisher) equilibrated with buffer A. The resin was washed with 10 mM imidazole in buffer A, then His-tagged protein was eluted in a stepwise fashion with increasing concentrations of imidazole (50 mM, 75 mM, 250 mM, 500 mM) in buffer A. Fractions containing pure protein were pooled, then concentrated and the buffer exchanged for 20 mM Tris (pH 7.5), 150 mM NaCl, 5% glycerol using Amicon Ultra-15 centrifugal filters before storage at −80°C.

### Enzyme assays

Measurements to determine kinetic parameters for hydrolysis, and kinetic parameters for inhibition were performed at ambient temperature in PBS (pH 7.4) containing 0.1 mg/mL bovine serum albumin, using a PowerWaveXS plate reader (BioTek).

Assays to determine Michaelis-Menten kinetic parameters were performed by mixing variable amounts of enzymes (depending on the enzyme-substrate pair), with serial dilutions of different BL substrates and continuously monitoring the reduction in absorbance associated with BL hydrolysis (28, 36, 40, 41). Kinetic parameters were determined by plotting initial rates of hydrolysis (*v*) as a function of substrate concentration [*S*] and fitting the data to equation 1 using GraphPad Prism,


(1) $$v=\frac{\left[Et]\times k_{cat}\times [S\right]}{K_{M}+[S]}$$

where [Et] is the enzyme concentration, *k*_cat_ the turnover number and *K*_M_ the Michaelis constant. For instances where substrate hydrolysis displayed linear kinetics (i.e. initial velocity of hydrolysis could not be saturated at obtainable substrate concentrations), *k*_cat_/*K*_M_ was estimated by fitting plots of velocity versus substrate concentration to equation 2, assuming [*S*] << *K*_M_ (29).


(2) $$v=\frac{k_{\text{cat}}}{K_{\text{M}}}\left[E][S\right]$$

Second-order rate constants (*k*_2_/*K*) denoting the inactivation efficiency of BLIs were determined by continuously measuring CENTA (100 µM) hydrolysis (28) in the presence of various concentrations of BLI. Progress curves were fit to equation 3 using GraphPad Prism, where *A*_i_ and *A*_0_ are the measured and basal absorbance, *v*_i_ and *v*_s_ are initial and final velocity, and *t* is time in seconds. *k*_2_/*K* was derived with equation 4. *K*_off_ was determined by the jump dilution method (24). *K*_d_ was estimated from the ratio between *k*_off_ and *k*_2_/*K*.


(3) $$A_{\text{i}}\text{=}A_{\text{0}}\text{+}v_{\text{s}}t\left(v_{\text{i}}\text{-}v_{\text{s}}\right)\text{[}\frac{\text{1-}{\text{e}}^{\text{-}\left(k_{\text{obs}}\right)t}}{k_{\text{obs}}}\text{]}$$

(4) $$k_{\text{obs}}\text{=}k_{\text{-2}}\text{+(}\frac{k_{\text{2}}}{K_{\text{I}}}\text{)}\frac{\text{[I]}}{\text{1+}\frac{\left[S\right]}{K_{\text{M}}}}$$

Affinities of CAZ, FEP and CTB for KPC variants were determined by competition assay (42), with CENTA as the reporter substrate. Enzyme was added to mixtures containing CENTA and various concentrations of CAZ, FEP or CTB, and the absorbance at 405 nm was continuously monitored. The inverse of the initial velocity was plotted against the corresponding inhibitor concentrations and the data fit to a linear equation to derive *K*_i app observed_ by dividing the y-intercept by the slope of the line. *K*_i app observed_ was corrected for substrate concentration and affinity using equation 5.


(5) $$K_{\text{i app}}\text{=}\frac{\text{[}K_{\text{I observed}}\text{]}}{\text{1+}\frac{\left[S\right]}{K_{\text{M}}\text{CENTA}}}$$

### Molecular modelling

Complexes between KPC-2^wt^ and KPC-2^D179Y^ with AVI, TAN or LED were generated using MOE software (43). Structures for KPC-2^wt^ (PDB: 5UL8) (44) and KPC-2^D179Y^ (PDB: 7TBX) (17) were imported into the software and prepared for docking using MOE QuickPrep. Molecular structures for AVI, TAN and LED were created in the MOE environment and energy minimized before covalent docking at the catalytic serine. Complexes with KPC-2^wt^ were validated by comparison with published co-structures for KPC-2-AVI (31) or KPC-2-TAN (32). For the KPC-2^D179Y^ complexes, models with the lowest binding energies are shown.
